# Supplementary material for: A filtering reconfigurable intelligent surface for interference-free wireless communications
Source: Nat Commun. 2024 May 7;15:3838. doi: 10.1038/s41467-024-47865-6 (PMC11076613; doi:10.1038/s41467-024-47865-6)
Supplement: Supplementary file 1 — Supplementary Information [file 41467_2024_47865_MOESM1_ESM.pdf]

Supplementary Information

A filtering reconfigurable intelligent surface for interference-free wireless communications

Jing Cheng Liang[†]*, Lei Zhang*[†]*, Zhangjie Luo*, Rui Zhe Jiang, Zhang Wen Cheng, Si Ran Wang, Meng Ke Sun, Shi Jin, Qiang Cheng*, and Tie Jun Cui**

Supplementary Note 1: Details on the Filtering RIS

The layered view of the RIS subarray is presented in **Supplementary Fig. 1**. It consists of four F4B (ε_r=3, tan δ=0.001) dielectric slabs, named slabs 1~4 from top to bottom. The upper layer of slab1 and the lower layer of slab4 are the parasitic patches of the antenna element. The power combining and dividing networks are on the upper layer of slab2 and the lower layer of slab3, respectively. The 2-bit phase shifter and the filter circuits are on the upper layer of slab2. The lower layer of slab2 and the upper layer of slab3 are metal grounds, which are used to avoid mutual interference of electromagnetic (EM) signals between the upper and lower spaces. A metallic via penetrates the ground and connects the output of the phase shifter on the top layer of slab2 and the input port of the retransmitted structure on slab3. Dielectric slabs 2 and 3 are bonded with 0.1 mm thick prepreg, Rogers 4450F (ε_r =3.7, tan δ=0.001).

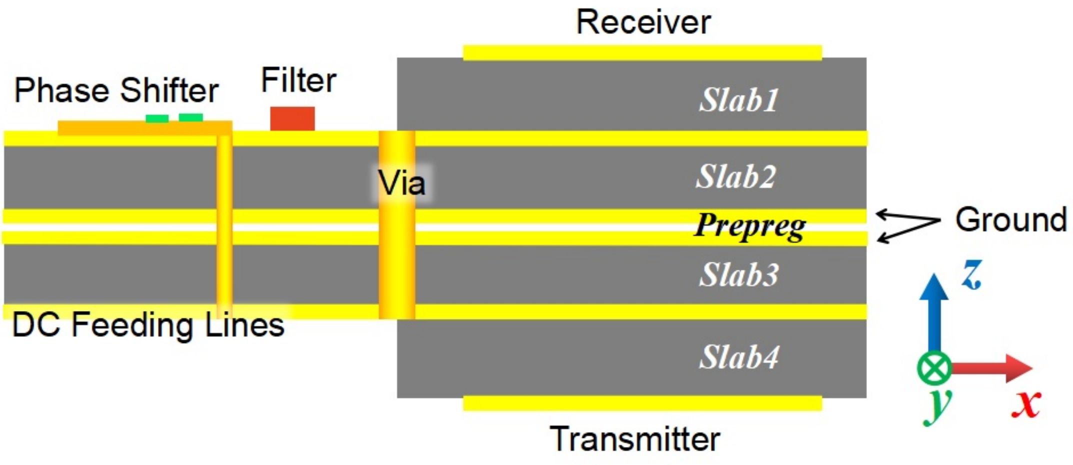

Supplementary Fig. 1. The layer view of the proposed filtering RIS.

Supplementary Note 2: Receiver and Transmitter

The receiver and transmitter in the RIS share the same configuration. They function to convert spatial waves to guided waves and vice versa. In this work, the receiver/transmitter contains 4×4 elements. The structure of the element is shown in **Supplementary Fig. 2**, consisting of three metal layers and the substrates between them. The bottom layer is the metallic ground. The middle layer is the excitation layer. The top layer is a metallic rectangular parasitic patch that couples EM energy from the middle layer. The element period is P_x=P_y=40 mm, which is less than the half wavelength at 3.5 GHz, thus avoiding the appearance of grating lobes. As shown in **Supplementary Fig. 3**a, the amplitude of the reflection coefficient at the excitation port of the element is less than -10 dB from 3.38 to 3.65 GHz. Besides, the gain of the element is between 6.9~7 dBi in the designed passband 3.4~3.6 GHz. Supplementary Fig. 3b depicts that the radiation efficiency of the 4×4 elements is greater than 97% and the radiation gain is greater than 15.8 dBi, which demonstrates that the receiver/transmitter enables efficient conversion efficiency between spatial waves and guided waves.

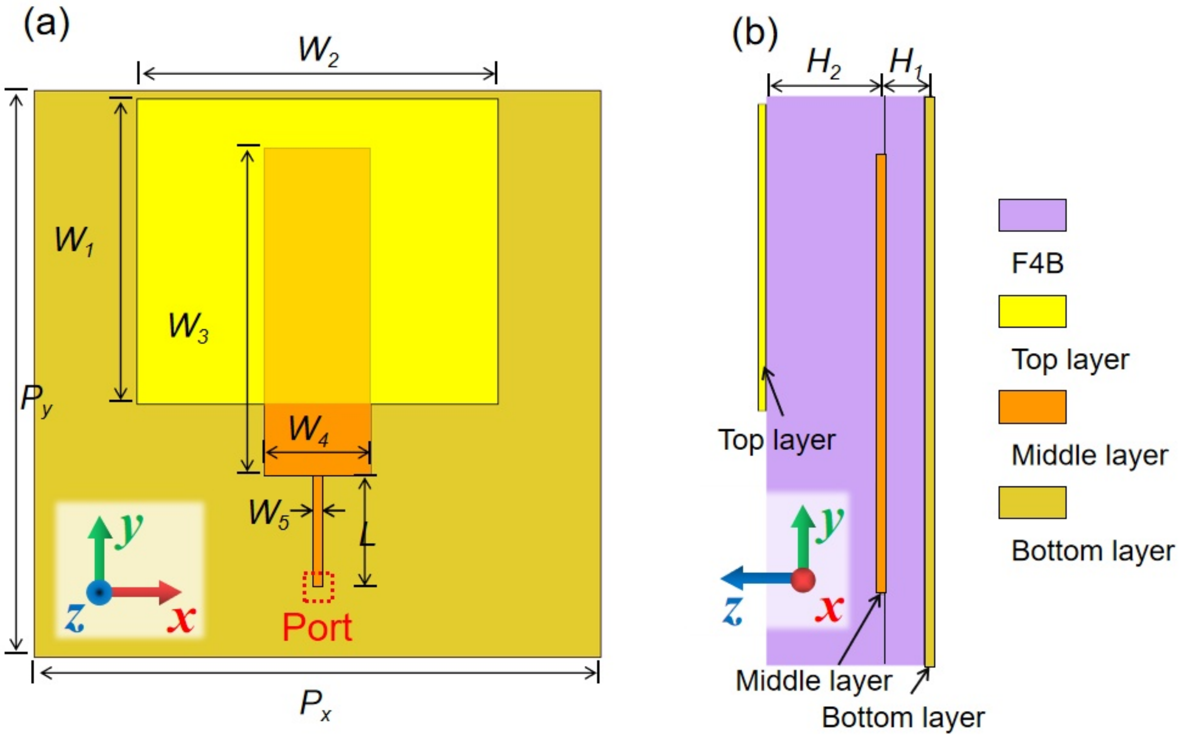

Supplementary Fig. 2. Sketch of the receiver/transmitter of each element. (a) Top view. (b) Side view. P_x=40 mm, P_y=40 mm, H_1=1.0 mm, H_2=3.0 mm, W_1=21.52 mm, W_2=25.50 mm, W_3=23.05 mm, W_4=7.54 mm, W_5=0.41 mm, L=7.83 mm.

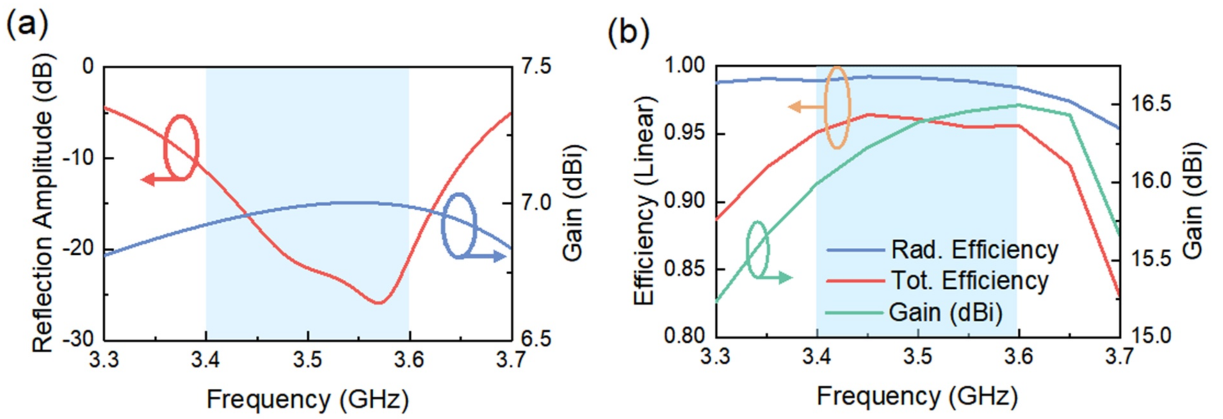

Supplementary Fig. 3. (a) Simulated reflection amplitude and gain of the receiver/transmitter element. (b) Simulated radiation efficiency and the gain of the receiver/transmitter with 4×4 elements.

Supplementary Note 3: Filter Chip

QPQ3500 (QORVO) filter chip is selected to implement the filtering function [1]. The single-input and single-output structure allows the filter to be connected directly to the microstrip line. The filter chip does not require any peripheral circuit such as direct-current (DC) feeding networks or isolators. It is compact (2 mm × 1.6 mm × 0.89 mm) and can be surface-mounted, which is more conducive to integration and miniaturization. **Supplementary Fig. 4** shows the reflection and transmission amplitude spectrum of the filter chip. The blue area depicts the passband (3.4~3.6 GHz) of this filter chip. The filter chip is well-matched in the operating band, with a reflection amplitude of less than -15 dB. The transmission amplitude spectrum shows a 1.5~2.3-dB insertion loss in the passband and a rejection of over 30 dB in the stopband. In full-wave simulations, the filter chip is equivalently modeled as a frequency-dependent resistor, whose values are shown in **Supplementary Table1**.

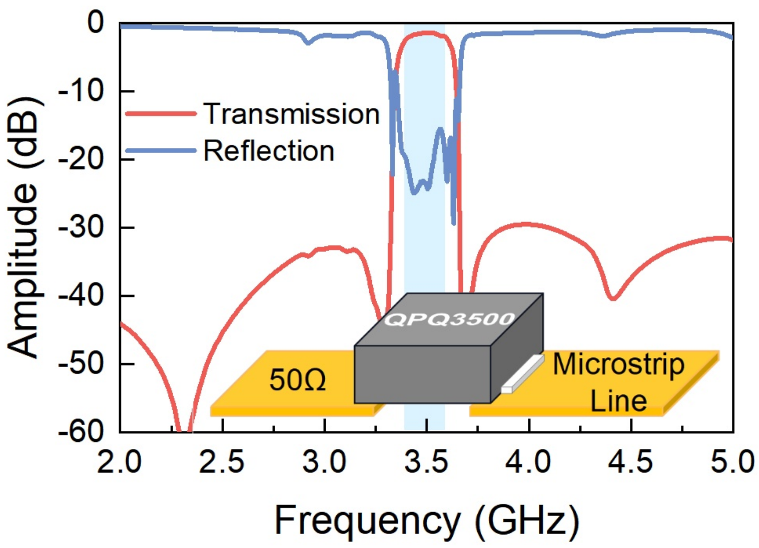

Supplementary Fig. 4. Simulated results of the transmission and reflection amplitudes of the filter chip.

Supplementary Table1. The equivalent resistance of the filter chip near the passband.

Frequency (GHz)	3.3	3.35	3.4	3.5	3.6	3.65	3.7
Equivalent Resistance (Ω)	18000	130	30.5	17	30.5	360	40000
Transmission Amplitude (dB)	-44.7	-7.11	-2.27	-1.35	-2.3	-14	-51

Supplementary Note 4**: Phase Shifter**

A 2-bit switched delay line phase shifter is designed to generate the four phase-coding states by switching different EM wave paths. The phase shifter features low insertion loss while maintaining the input/output matchings [2], [3]. **Supplementary Fig. 5**a shows the structure of the 2-bit phase shifter, and Supplementary Fig. 5b presents the equivalent circuits. The path of the guided wave is selected by switching the "on" and "off" states of the four pairs of PIN diodes, which can be combined to produce four different phase states (states0~3) of 0°, 90°, 180°, and 270°, as shown in Supplementary Fig. 5c.

To minimize the insertion loss of the phase shifter, PIN diodes with low series resistance (SMP1321-040LF, Skyworks) are employed. The PIN diode in the "off" state can be modeled as a series connection of a 15 Ω resistor and a 0.15 pF capacitor, which produces an isolation of 9.7 dB. By connecting two PIN diodes in series, the isolation is increased to 15 dB when both PIN diodes are in "off" state. The PIN diode in the "on" state can be equivalent to a series connection of a 0.5 Ω resistor and a 0.3 nH inductor. The insertion loss is about 0.3 dB when both series-connected PIN diodes are in "on" state. **Supplementary Fig. 6**a shows that the insertion losses for four coding states are around 0.8 dB in the designed passband 3.4~3.6 GHz. The phase difference between adjacent states is about 90° in the passband, as shown in Supplementary Fig. 6b.

In addition, corresponding DC feeding lines are designed to provide the controlling signals for the PIN diodes in the phase shifter, as shown in Supplementary Fig. 5a. The DC feeding lines are connected to both sides of the PIN diodes through metallic vias. Radio-frequency (RF) chokes (muRata-LQG15HS1ONG02) with a self-resonant frequency of 3.4 GHz are mounted on the DC feeding lines to suppress additional resonance introduced by the DC feeding lines. At the output of the phase shifter, the signal is transmitted to the microstrip line on the slab3

through a metallic via, as shown in Supplementary Fig. 5a.

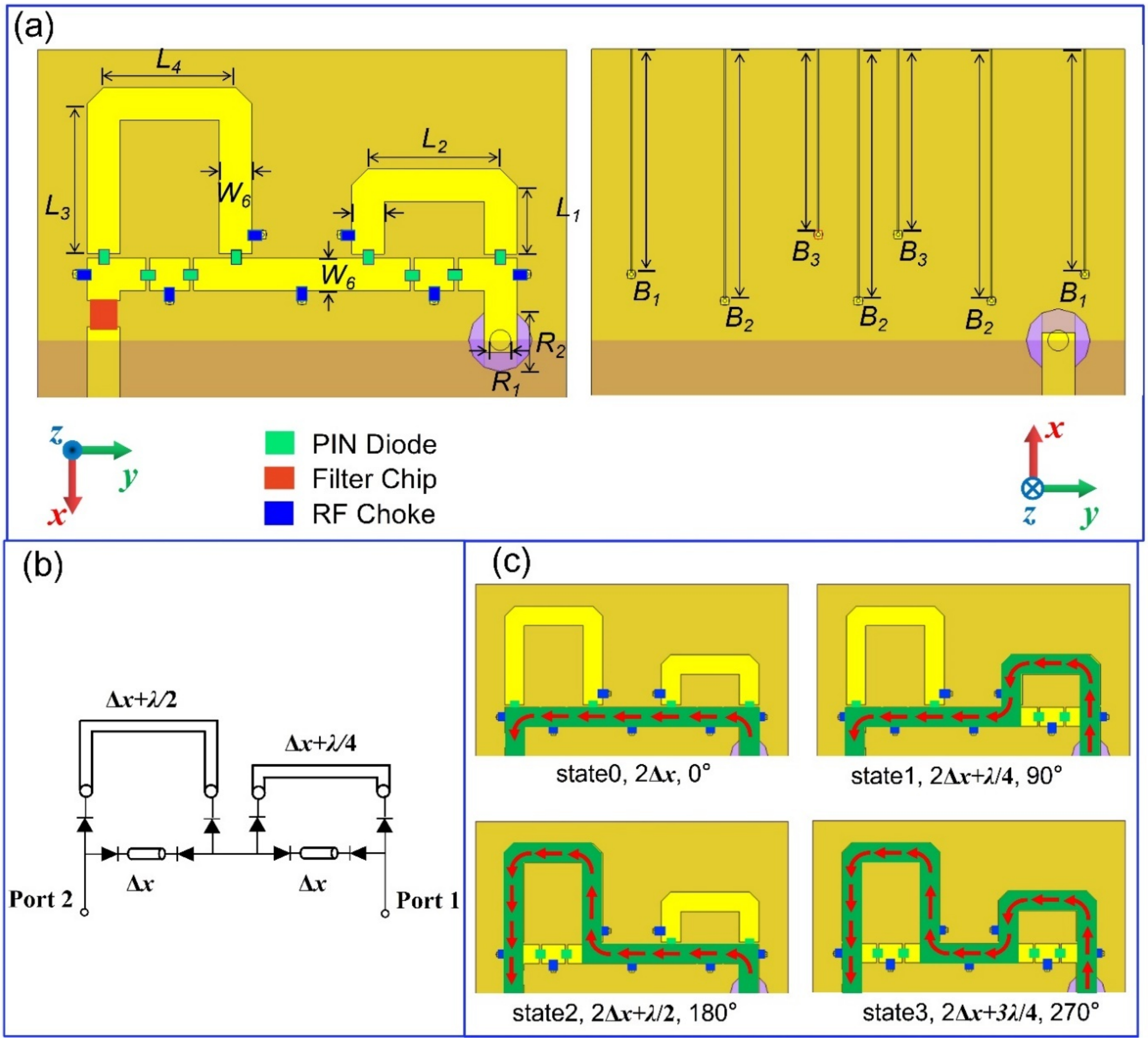

Supplementary Fig. 5. The 2-bit switching delay line phase shifter. (a) Top and bottom views. (b) Equivalent circuit diagram. (c) Switchable current paths of the phase shifter in four coding states (states0~3). L_1=5.21 mm, L_2=10.03 mm, L_3=11.41 mm, L_4=10.03 mm, R_1=1.56 mm, R_2=4.80 mm, B_1=16.85 mm, B_2=18.85 mm, B_3=13.85 mm, W_6=2.48 mm.

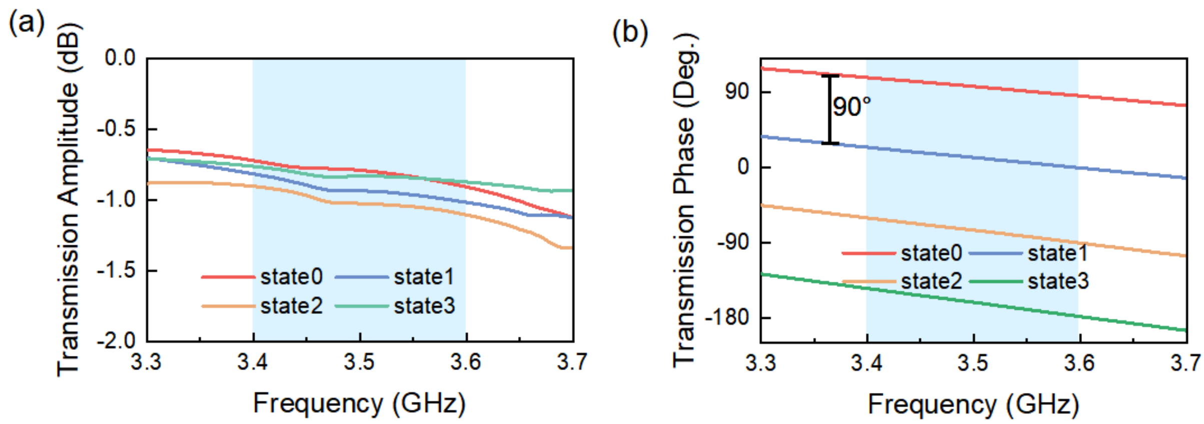

Supplementary Fig. 6. Simulated results of the transmission coefficients of the 2-bit phase shifter. (a) The amplitude. (b) The phase.

Supplementary Note 5: Loss Analysis

The main source of the RIS losses is analyzed as follows.

1. Conversion loss between the spatial waves and the guided waves. According to Supplementary Fig. 3, the conversion efficiency of the 4×4 receiver/transmitter is 95%-97%, which means that the conversion loss of the whole panel is between 6% and 10%. This loss can be reduced by optimizing the radiation properties of the receiver and transmitter and improving the impedance match between the receiver/transmitter and the circuits.

2. Insertion loss of the filter chips. As depicted in Supplementary Fig. 4, the filter chip achieves the stopbands with over 30 dB rejection, but it also has an insertion loss of 1.5-2.3 dB. This implies that about 29%-42% of the power is attenuated.

3. Insertion loss of the phase shifters. According to Supplementary Fig. 6a, the loss is around 0.7-1.0 dB, meaning that about 15%-21% of the power is attenuated. It is primarily caused by the ohmic loss of the PIN diode.

The above discussions reveal that the loss caused by the filter chip contributes significantly to the RIS's overall insertion loss. It occurs at the expense of the sharp frequency selection and high rejection in the stopbands. This can be mitigated by using low-loss filter chips. Moreover, the loss caused by the phase shifters can be reduced by selecting PIN diodes with superior switching characteristics. Inspired by the current amplifying RIS[4]-[6], we can further integrate low-noise amplifiers into the functional module to compensate for the loss.

Supplementary Note 6: Performance of the Subarray under Oblique Incidences

Simulations and measurements are conducted to analyze the transmission features of the proposed RIS under oblique incidences. The amplitude spectra under the transverse-magnetic (TM) mode with incidence angles ranging from 0° to 60° are studied for starters. The simulated and measured results with Coding state 0 are depicted in **Supplementary Figs. 7**a and 7b, respectively, which agree quite well with each other. As the incident angle increases, the passband frequency remains stable, with the transmission amplitudes larger than those in the stopbands by 20 dB. The results with the other three coding states are similar. Under the TM incidence, the transmission amplitudes and phases at 3.5 GHz that vary with the incident angles for the four coding states are shown in Supplementary Figs. 7c and 7d, respectively. It can be seen that the amplitudes vary between -2 and -4 dB. The phase differences between the four coding states are nearly 90°. These results indicate the stable performances of the proposed RIS under TM oblique incidences.

Under the transverse-electric (TE) oblique incidence, both the filtering performance and the transmission amplitude degrade as the incident angle increases to 20°, as shown in **Supplementary Figs. 8**a and 8b, respectively. This is because the in-phase condition of the four elements in the subarray deteriorates under the TE oblique incidence. The amplitudes and phases of the transmission coefficient versus the incidence angle at 3.5 GHz are shown in Supplementary Figs. 8c and 8d, respectively. Putting aside the amplitude decline, the phase difference between the four coding states remains 90° as the incident angle varies.

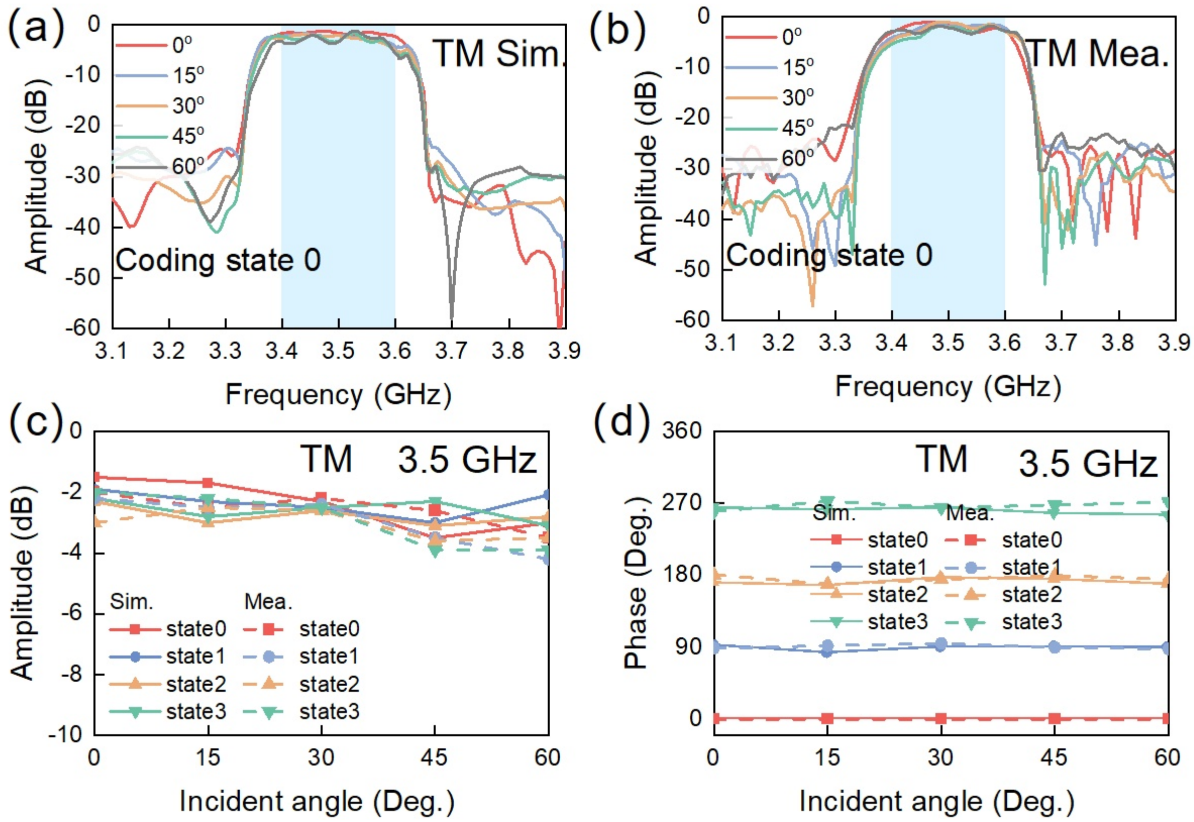

Supplementary Fig. 7. The transmission coefficients under TM oblique incidences. (a) Simulated and (b) measured transmission amplitude spectra with Coding state 0 with incident angles from 0° to 60°. Transmission (c) amplitudes and (d) phases at 3.5 GHz versus the oblique incident angles with the four coding states.

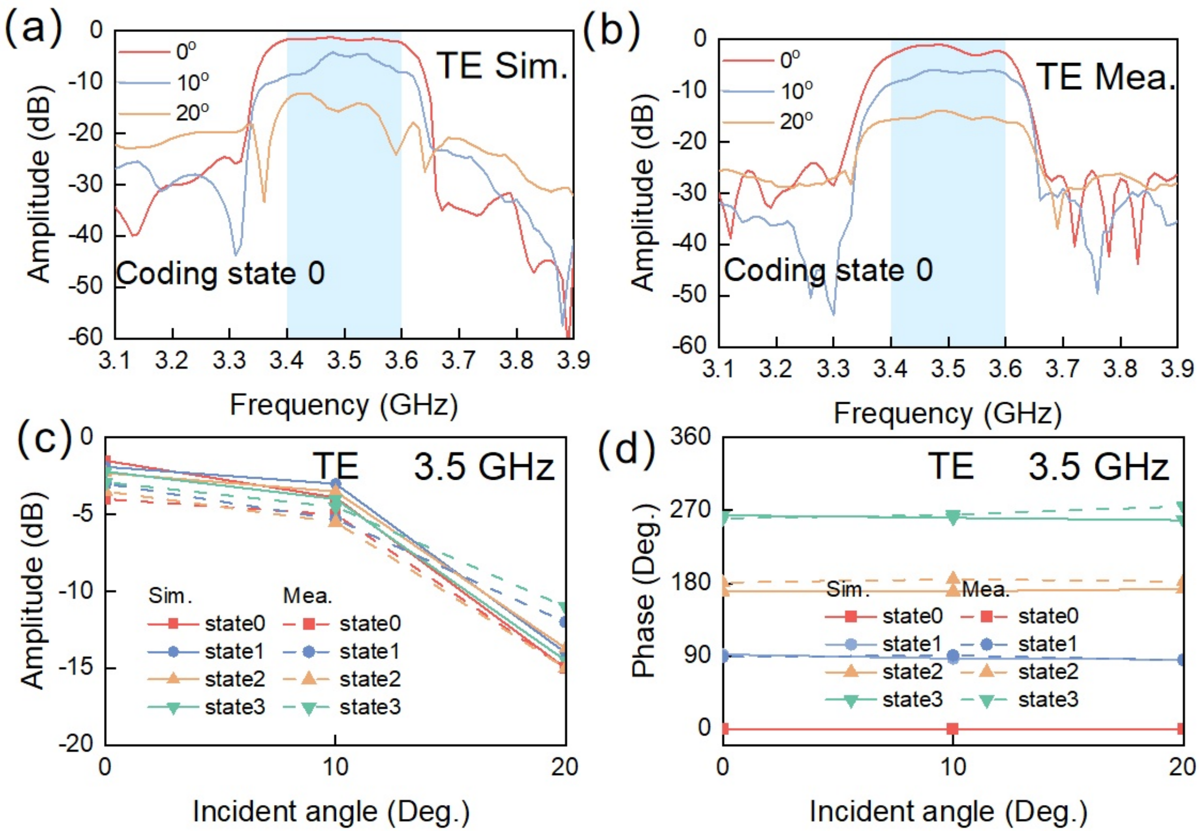

Supplementary Fig. 8. The transmission coefficients under TE oblique incidences. (a) Simulated and (b) measured transmission amplitude spectra with Coding state 0 with incident angles from 0° to 20°. Transmission (c) amplitudes and (d) phases at 3.5 GHz versus the oblique incident angles with the four coding states.

Supplementary Note 7: Design of the Single RIS Element and 2D Beamforming

We have designed a new filtering RIS element following the same principle, which has a pair of transmitter and receiver and a functional module for filtering and phase shifting. The structure of the element and the structural parameters are shown in **Supplementary Figs. 9**a and 9b, and its simulated transmission amplitudes and phases are shown in Supplementary Figs. 9c and 9d, respectively. The transmission amplitudes of the four phase coding states range from -1.2 to -4.2 dB between 3.4 and 3.6 GHz. On the two sides of the passband, rejections of 30 dB in the stopbands are obtained. In this operating bandwidth, stable 90° phase differences are exhibited between the curves. The Q factor of this filtering RIS element is 14.4, and the K20dB is 1.4. which are extremely close to the results of the subarray discussed in the main text.

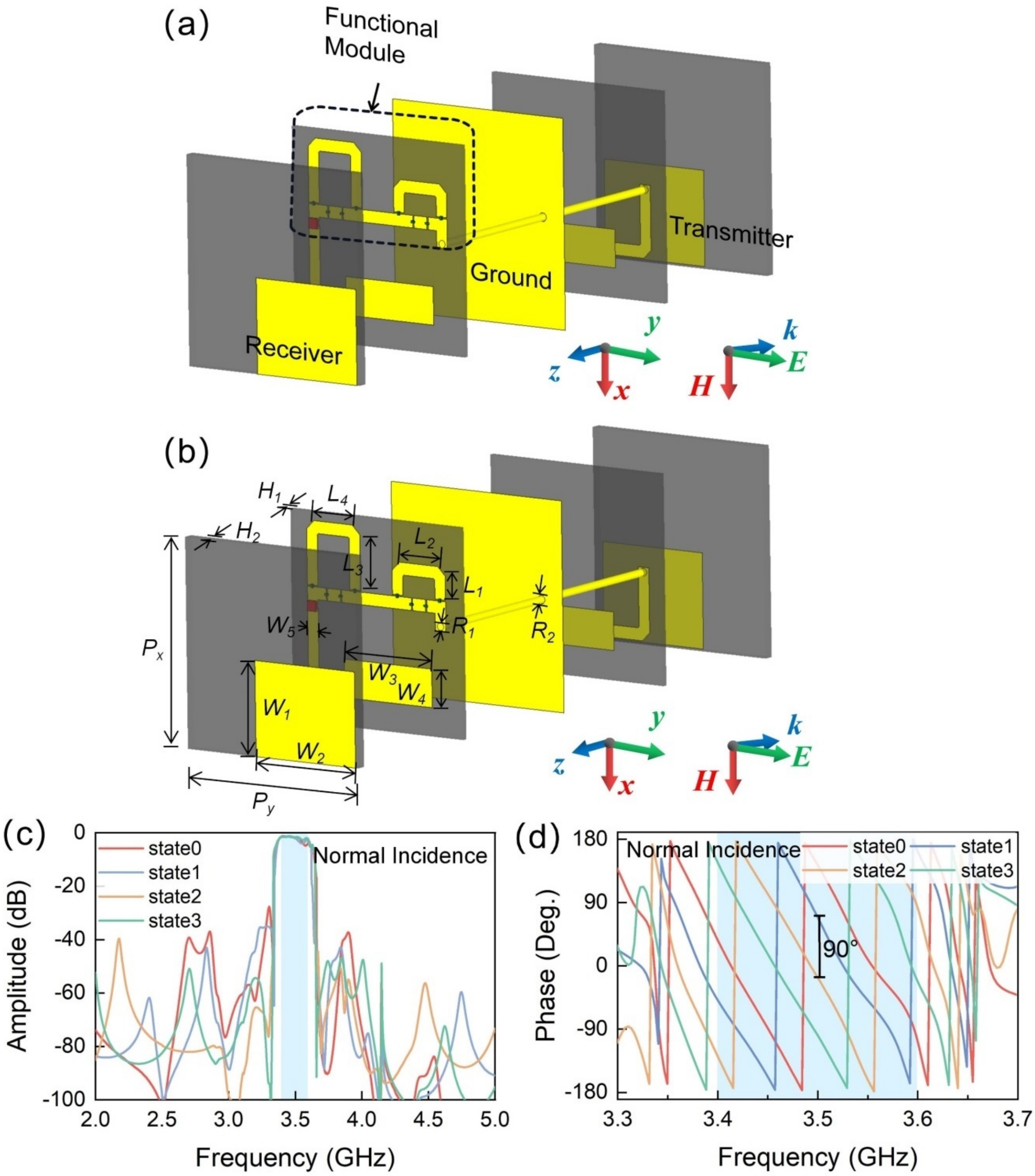

Supplementary Fig. 9. The newly designed single filtering RIS element. (a) Configuration. (b) Structural parameters. P_x=41.39 mm, P_y=40 mm, H_1=1.0 mm, H_2=2.0 mm, W_1=18.78 mm, W_2=23.38 mm, W_3=20.56 mm, W_4=7.53 mm, W_5=2.45 mm, L_1=5.21 mm, L_2=10.03 mm, L_3=11.41 mm, L_4=10.03 mm, R_1=1.52 mm, R_2=1.72 mm. (c) and (d) The simulated transmission coefficients. (c) Amplitude and (d) phase spectra of the four phase coding states (States 0-3) under the normal incidence.

We further studied the transmission coefficients of this RIS element under TM and TE oblique incidence ranging from 0° to 60°. As examples, the amplitude spectra with Coding state 0 are depicted in **Supplementary Fig. 10**a. It is observed that the amplitude spectra are minimally affected by the incident angle. The results with the other three coding states are

similar. The passband frequency remains stable, and rejections of more than 20 dB are observed outside the passband. We then focused on the transmission amplitudes and phases at 3.5 GHz that vary with incident angles for the four coding states, as shown in Supplementary Figs. 10b and 10c, respectively. The amplitudes under the TM incidences are quite stable even when the angle is as large as 60°; the amplitudes under the TE incidences start to deteriorate when the angle is about 30°, and end up to about -5 dB when the angle is 60°. The Q factors and K20dB values are shown in Supplementary Figs. 11a and 11b, respectively. It is observed that the Q factors are higher than 14 and the K20dB values remain almost unchanged in all conditions, indicating stable filtering responses under both the TM and TE oblique incidences. For the transmission phase performances, stable 90° shiftings are exhibited under both TM and TE incidences, as displayed in Supplementary Fig. 10c.

To verify the 2D beamforming ability, we constructed an RIS with 10×10 elements and simulated the far-field patterns of transmitted signals when it is normally illuminated by plane waves at 3.5 GHz. The results are illustrated in Supplementary Fig. 12, showing that by carefully adjusting the phases of the elements, the beam can be tilted within wide angle ranges of ±63° and ±64° on the *xoz*- and *yoz*-planes, respectively. The viable way to realize the 2D beamforming using the proposed technique is thus proved.

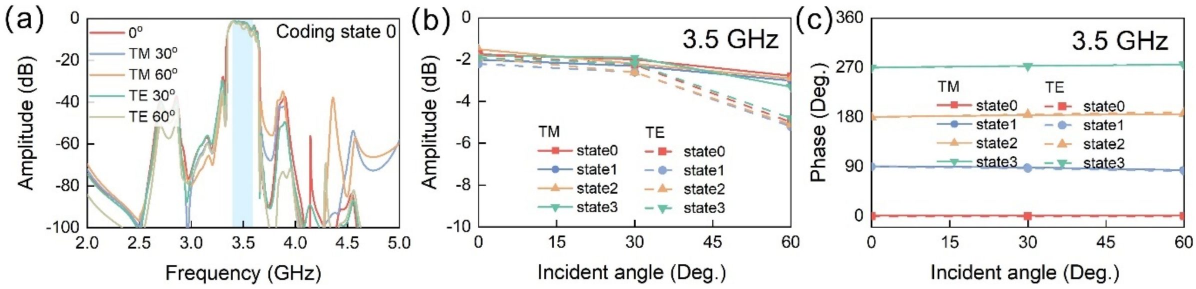

Supplementary Fig. 10. The simulated transmission properties of the newly designed single RIS element. (a) The amplitude spectra with Coding state 0 under the TM and TE mode with incident angles from 0° to 60°. Transmission (b) amplitudes and (c) phases at 3.5 GHz versus the oblique incident angles with the four coding states.

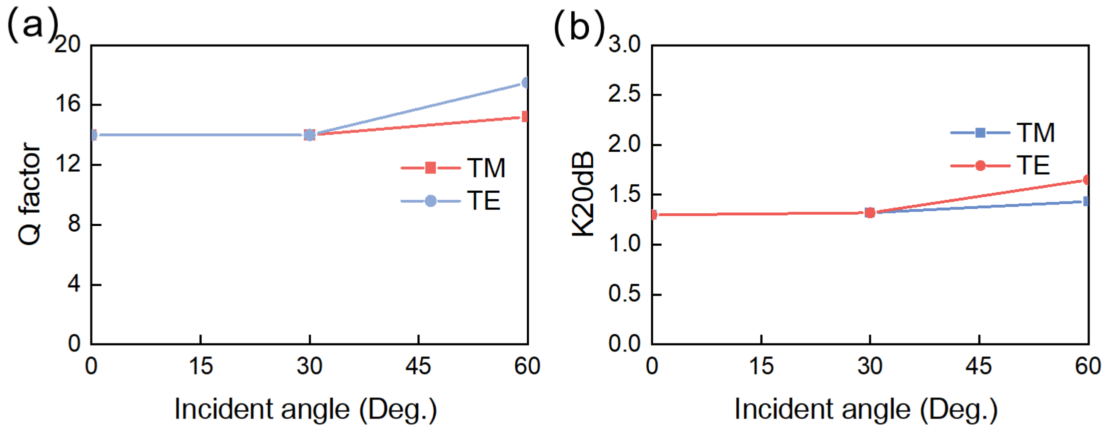

Supplementary Fig. 11. The Q factor and K20dB of the newly designed single filtering RIS element versus the incident angles under (a) TM and (b) TE modes.

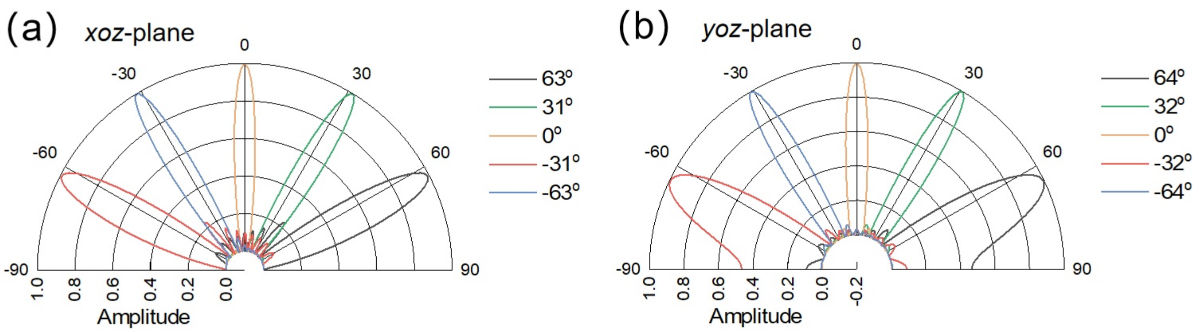

Supplementary Fig. 12. The simulated far-field patterns of the RIS with 10×10 newly designed single elements. Each RIS element has a pair of transmitter and receiver and a functional module for filtering and phase shifting. (a) *xoz*-plane. (b) *yoz*-plane.

Supplementary Note 8: Design of a Millimeter-Wave Filtering RIS

To compare the proposed technique with other RISs at higher frequencies, we have designed a mmWave filtering RIS using the same method, whose structure is shown in **Supplementary Fig. 13**a. A filtering chip (MMCB2528G5T-0001A3, TDK)[7] is employed here. We conduct field-circuit cosimulations to study the features of this structure, and the results are illustrated in Supplementary Figs. 13b and 13c. The transmission amplitudes of the four coding states range from -3.3 to -6.3 dB between 26.4 GHz and 30.4 GHz. On the two sides of the passband, a 30-dB rejection in the stopbands is obtained. In the operating bandwidth, stable 90° phase differences are exhibited between the curves. The Q factor for this mmWave filtering RIS is 7.1.

The results are compared with Ref. [8] in **Supplementary Table2**. Compared with Ref. [8], our mmWave RIS has a larger BW/BW3dB value. The rectangle coefficient K20dB of our

mmWave RIS is 1.4, indicating steeper transitions on the two edges of the transmission curve and thus a better filtering effect than the reference. These results prove the stronger filtering and phase-tuning properties of the proposed RIS in this manuscript.

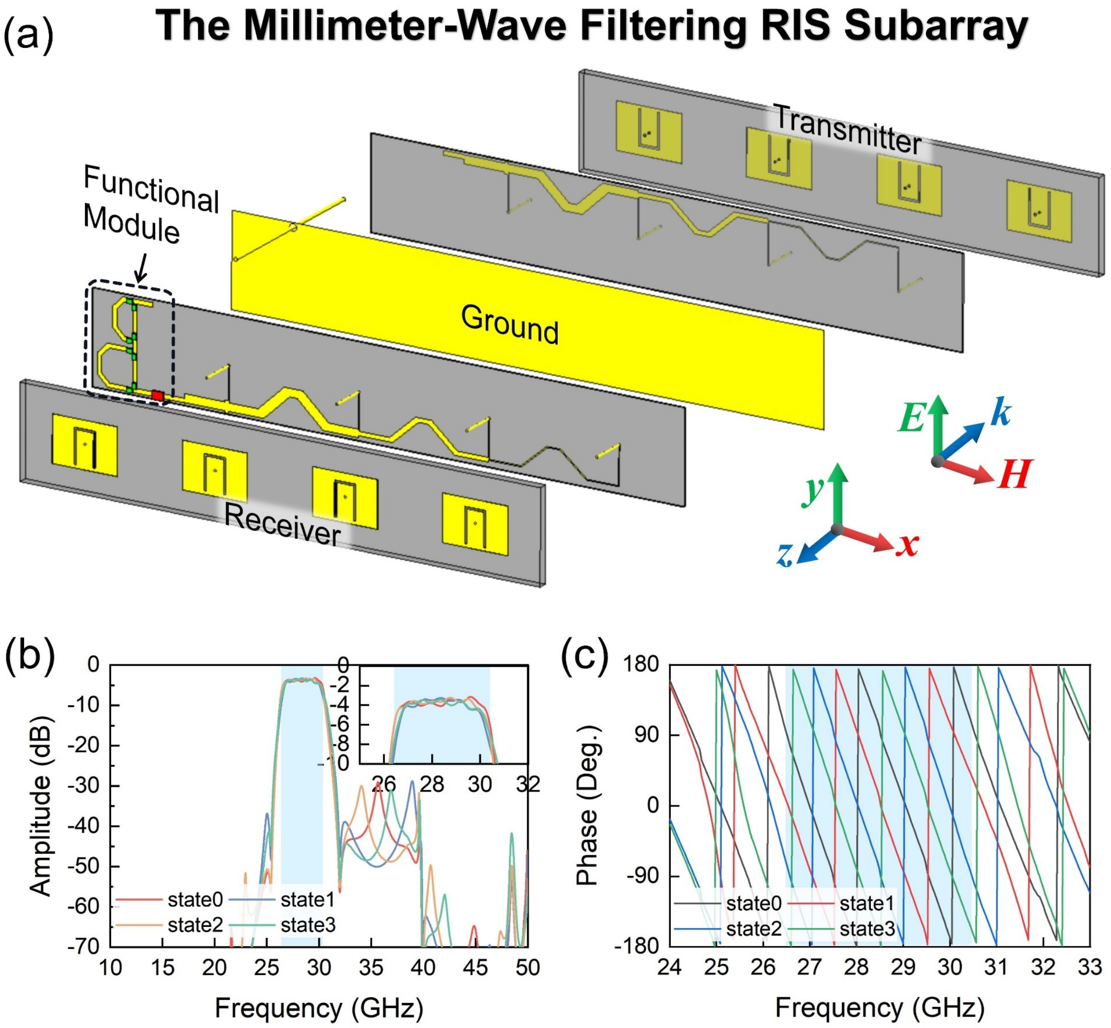

Supplementary Fig. 13. The millimeter-wave filtering RIS. (a) Configuration. The transmission coefficients obtained from the cosimulations. (b) Amplitude and (c) phase spectra of the four digital states (States 0-3).

Supplementary Table2. Comparison between the mmWave RIS in this work and the one in Ref. [8].

Ref.	Type	f_0 (GHz)	Operating BW (GHz)	BW3dB (GHz)	BW20dB (GHz)	BW/ BW3dB	Q factor	K20dB
**mmWave RIS (Simulated)**	**Rx. - Fun. - Tx.**	**28.5**	**3.6 (12.6%)**	**4.0 (14.0%)**	**5.6 (19.6%)**	**90.0%**	**7.1**	**1.4**
[8]	Rx. - Tx.	32.0	4.3 (13.4%)	4.9 (15.3%)	10 (31.2%)	87.8%	6.5	2.0

f_0, center frequency; BW, bandwidth; BW*n*dB, *n* dB bandwidth; Q factor, quality factor, the ratio f_0/BW3dB; K20dB, rectangle coefficient, the ratio BW20dB/BW3dB; Rx., receiver; Fun., functional module; Tx., transmitter.

Supplementary Note 9: Implementation of Wireless Communication Experiment

Supplementary Figs. 14a through 14e present the constellation diagrams, recovered pictures, and experiment photographs of the five cases in the wireless communication measurement. Horn antennas are employed as the transmitting and receiving antennas to eliminate uncontrollable multipath effects. The constellation diagrams in Cases 3 and 4 are cluttered, and the pictures are not well recovered.

Supplementary Figs. 15a through 15e depict the same wireless communication setup as that in Supplementary Fig. 14, except that the horn antennas are replaced by two pairs of custom-built patch antennas. One pair of patch antennas operate at 3.5 GHz, and the other pair work at 3.9 GHz. Their gains are 5.5 dBi and 5.9 dBi, respectively, and the input reflection coefficients at their working frequencies are less than -15 dB, as shown in **Supplementary Fig. 16**. **Supplementary Fig. 17** presents the SNR versus the radiated power in the five cases at 3.5 and 3.9 GHz. At 3.5 GHz in Supplementary Fig. 17a, the SNR values in Cases 3 and 4 are larger than those in Case 5, confirming the beamforming feature of the RIS. In Supplementary Fig. 17c, the SNR values in Cases 3 and 4 are higher at 3.5 GHz than those at 3.9 GHz, demonstrating the filtering effect of the RIS. The experiments demonstrate the impressive frequency-selecting and beam-steering characteristics of the filtering RIS.

We also perform wireless communication experiments outdoors where the absorbing screen around the RIS is replaced with a brick wall. The thickness of the wall is about 23 cm. The pictures are offered in **Supplementary Figs. 18a** through 18e. The transmitting and receiving antennas are still the horn antennas, which are placed on the two sides of the wall. It should be noticed that almost identical results are obtained with or without the metallic plate (Cases 1

and 2, respectively), which is attributed to the poor shielding effect of the wall and the small size of the plate. Then, we put the RIS in the window in Cases 3, 4, and 5, and the results are given in Supplementary Figs. 18c through 18e. The blocking effect of the RIS on the 3.9 GHz signals can be barely observed. At 3.5 GHz, no matter how we move the receiving antenna or adjust the coding sequence, the signals can still be received and recovered well. These results are quite different from those with the absorbing screen, and they are attributed to the small size of the RIS and the relatively strong transmission property of the wall. It is believed that the performance will improve by using an RIS with a larger aperture.

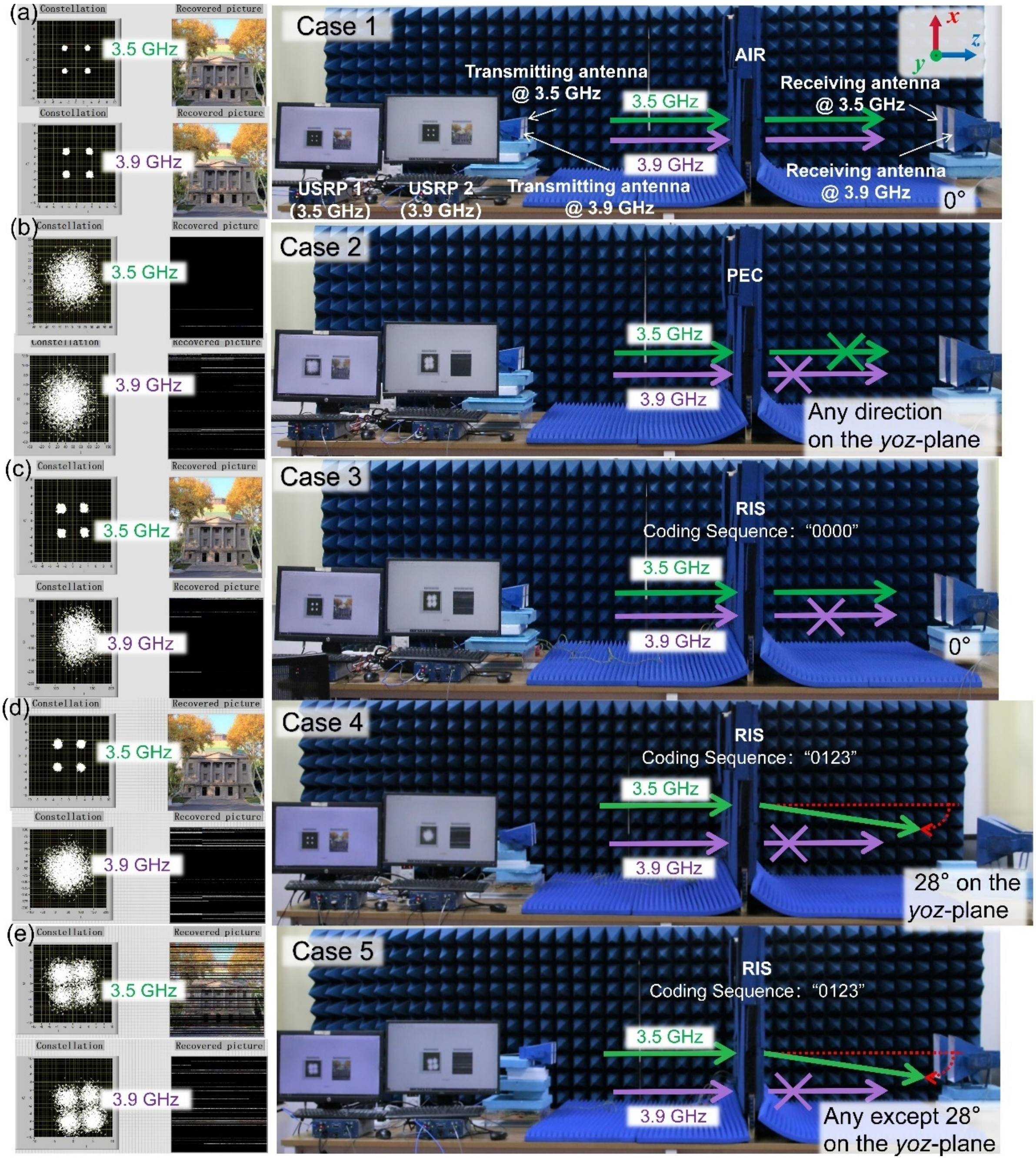

Supplementary Fig. 14. Pictures of the five experiments in the realistic wireless communication scenario. (a)-(e) Five cases with different frequencies and coding sequences.

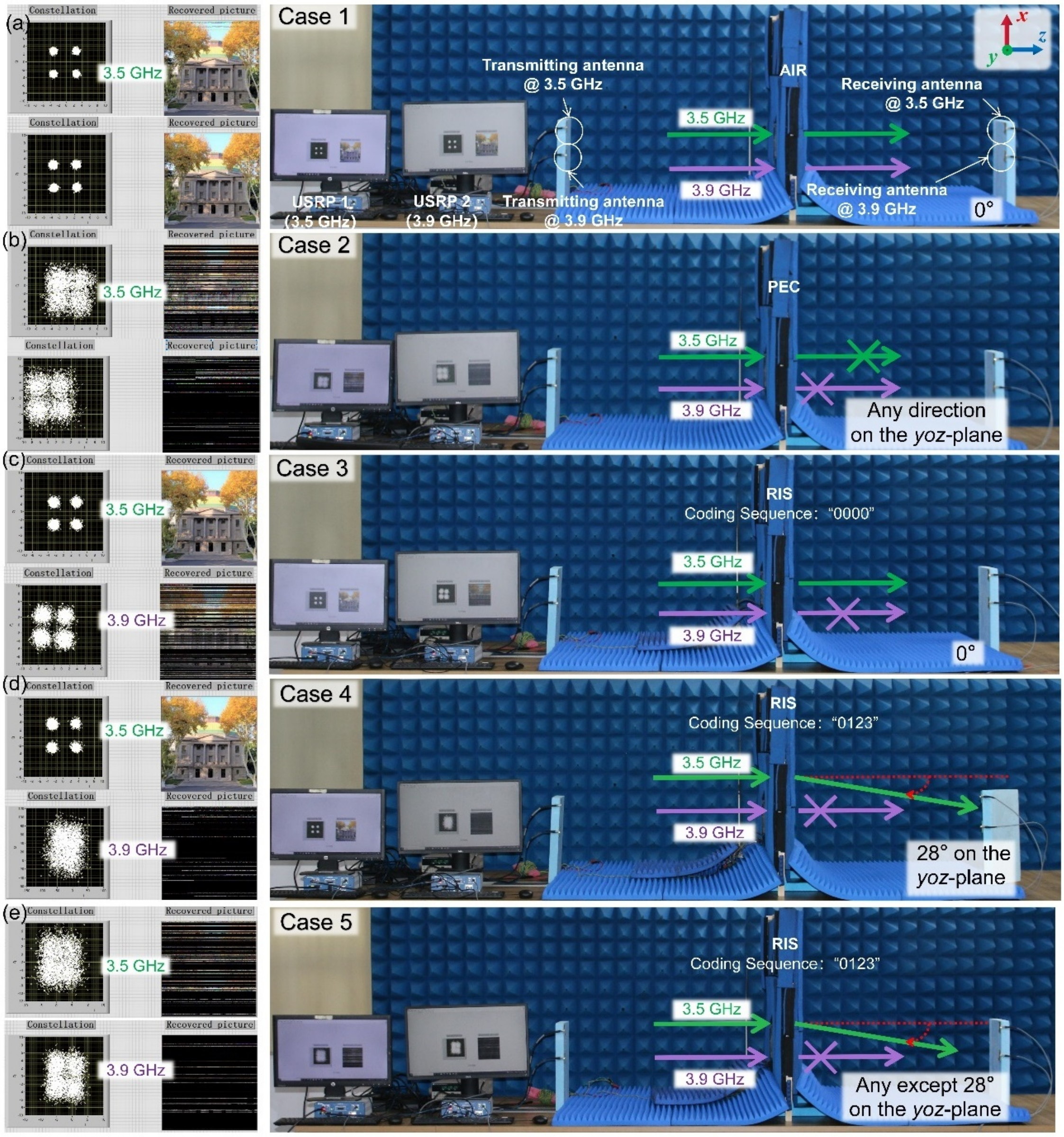

Supplementary Fig. 15. Five cases in the realistic wireless communication scenario using the patch antennas. (a)-(e) Five cases with different frequencies and coding sequences.

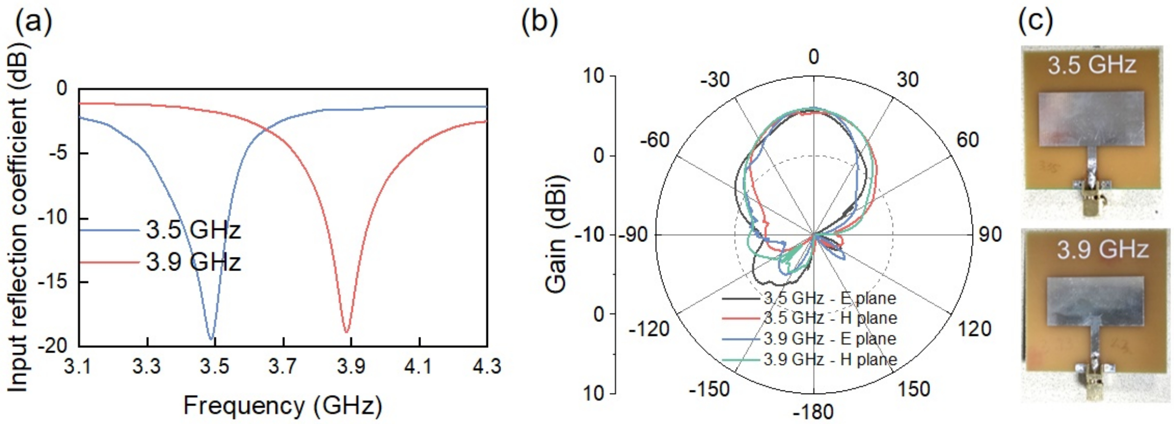

Supplementary Fig. 16. (a)The input reflection coefficient, (b) the measured gain, and (c) the photographs of the patch antennas (3.5 GHz and 3.9 GHz).

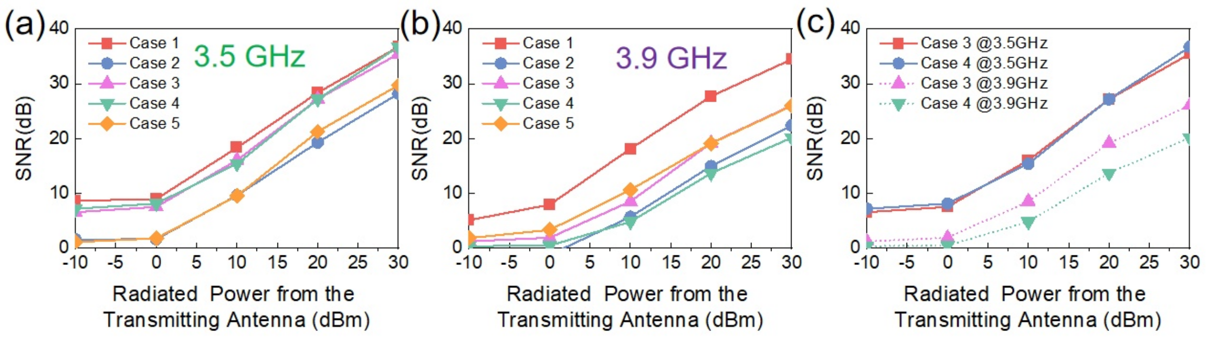

Supplementary Fig. 17. The SNR spectra as a function of the radiated power using the patch antennas in the five cases at (a) 3.5 GHz and (b) 3.9 GHz. (c) SNR in Cases 3 and 4 with the radiated power of 0 dBm.

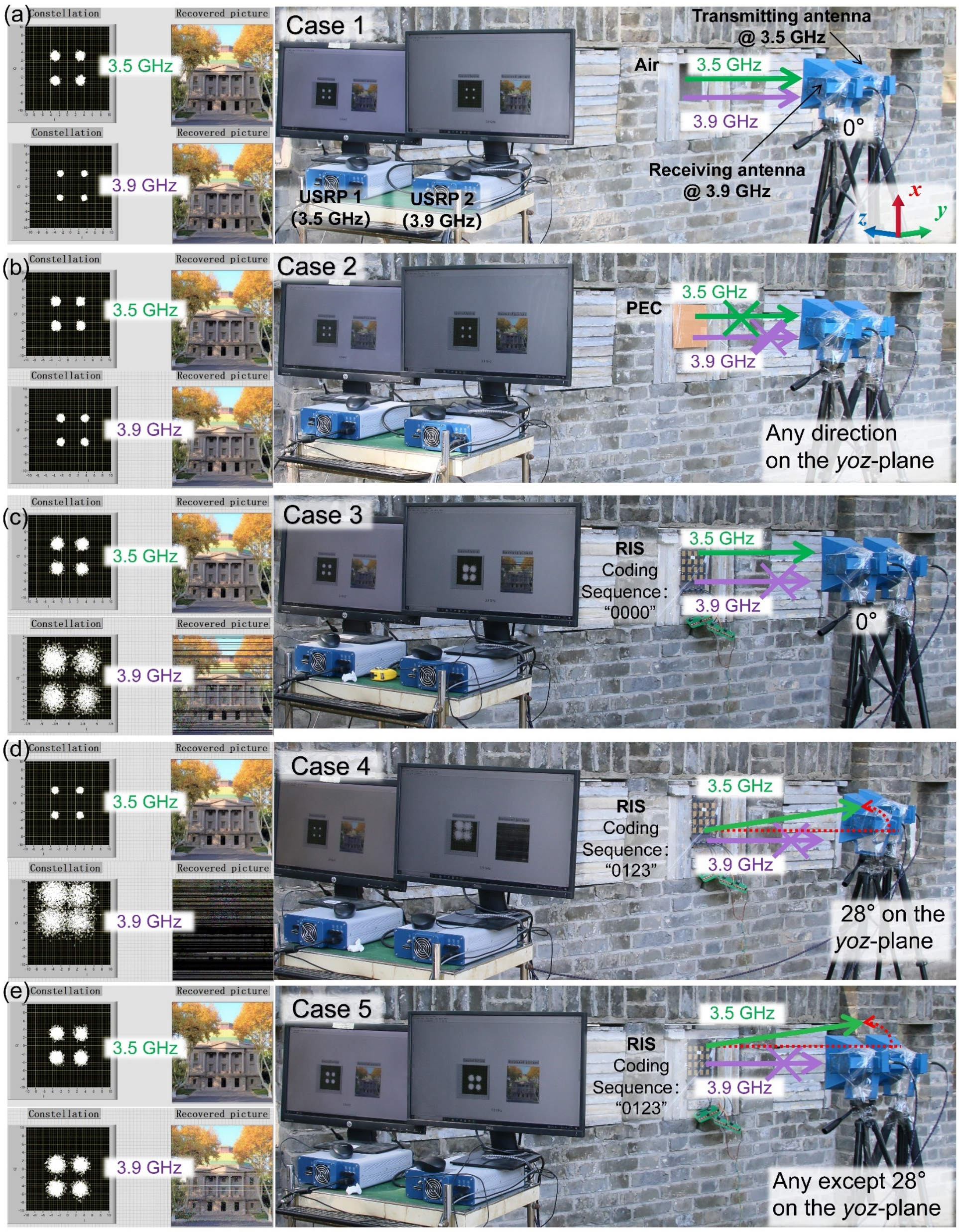

Supplementary Fig. 18. Five cases in the realistic wireless communication scenario where the absorbing screen around the RIS is replaced with a brick wall. The two transmitting horn antennas are on the other side of the wall, hence not shown in the pictures. (a)-(e) Five cases with different frequencies and coding sequences.

Supplementary Note 10: Comparison Between the Proposed RIS and Frequency Selective Surfaces (FSSs)

Generally, there are two types of FSSs, bandpass FSSs [9]-[11] and bandstop FSSs [12][13]. The bandpass FSSs allow signals around the center frequency to pass through, while the bandstop FSSs operate oppositely. Here the proposed RIS is compared with the bandpass FSSs.

Two main advantages are demonstrated by the proposed RIS over the bandpass FSSs. Firstly, it shows a much stronger frequency selectivity via a single-layer configuration. This can be proved by the comparison made in **Supplementary Table3**, showing that the K20dB value of the RIS is almost the same as that of the stacked 5-layer FSSs, but the RIS outperforms them in terms of BW3dB, BW20dB, and Q factor.

The second advantage held by the proposed RIS over the FSSs is the ability for beam manipulation. As discussed in references [14-16], RISs have shown significant potential in indoor wireless systems. Compared to the passive FSSs, the beamforming function of the RIS can optimize the wireless channel, thereby increasing the signal strengths in target directions and potentially enhancing the interference immunity for indoor wireless communications. Additionally, it is believed that RISs on interior walls are of great help in circumventing indoor blockages and extending coverage of cell signals in the sub-6G frequency bands [14-18].

Supplementary Table3. Comparison between the proposed RIS and the FSSs in previous studies.

Ref.	Configuration	Filtering type	f_0 (GHz)	BW3dB (GHz)	BW20dB (GHz)	Q factor	K20dB
**This work**	**Rx. - Fun. - Tx.**	**Bandpass**	**3.5**	**0.25 (7.1%)**	**0.32 (9.1%)**	**14**	**1.3**
[9]	Single Layer FSS	Bandpass	3.82	0.5 (13.1%)	>2.5 (65.4%)	7.64	>5
[10]	Stacked 5-Layers	Bandpass	5.4	0.9 (16.7%)	1.1 (20.4%)	6.0	1.2
[11]	Stacked 5-Layers	Bandpass	5.4	0.7 (13.0%)	0.9 (16.7%)	7.7	1.3
[12]	Single Layer FSS	Bandstop	5.8	-	-	-	-
[13]	Single Layer FSS	Bandstop	2.85	-	-	-	-

f_0, center frequency; BW*n*dB, *n* dB bandwidth; Q factor, quality factor, the ratio f_0/BW3dB; K20dB, rectangle coefficient, the ratio BW20dB/BW3dB; Rx., receiver; Fun., functional module; Tx., transmitter.

Supplementary Note 11: Comparison Between the Proposed RIS and the Conventional Repeaters/Relays

Compared with a relay, the advantages of the RIS are briefly discussed as follows.

1. As presented in Ref. [19], "the wireless environment is modeled as an exogenous entity that cannot be controlled, but only adapted to." The utilization of relays is one of the common approaches to capitalize on the uncontrollable wireless environment. However, relays are active devices that need dedicated power sources for operation. They are equipped with active electronic components, such as analog-to-digital converters (ADCs), digital-to-analog converters (DACs), mixers, and power amplifiers for transmission. Therefore, the deployment of relays is costly and power-consuming.

In contrast, the RIS provides a promising solution to shape the wavefront of EM waves and thus make the wireless environment customizable. Different from a relay, it does not need the active components mentioned above. It is mainly composed of a dielectric substrate, metallic patches, and functional microstrip lines. Its reconfigurable properties are achieved by controlling the switching status of PIN diodes through simple direct-current (DC) biasing wires. Therefore, the RIS has a much lower power consumption and complexity. Only power supplies for the controlling module are required, resulting in significant energy savings.

2. As presented in Ref. [19], full-duplex (FD) relays introduce high loop-back self-interference because of the concurrent transmission and reception of signals. Additionally, they generate co-channel interference at the destination, since relays and transmitters emit different information on the same physical resource. On the contrary, the proposed RIS does not contain any non-reciprocal components and therefore supports an FD mode of operation at a very low cost.

3. The active electronic components used in relays are responsible for the presence of additive noise. In amplify-and-forward (AF) relaying, the noise is also amplified at the relays [9]. The RIS does not contain any amplifiers at the current stage, so its performances are not affected by the additive noise.

4. Ref. [20] demonstrates that the decode-and-forward (DF) relays are more sensitive to electromagnetic interference (EMI), which may arise from a variety of causes, e.g., other (single or multiple) transmitting devices and/or natural background radiation. The authors

believed that “RIS-aided communications are more resilient to EMI” because of the spatially filtering capabilities of RISs. Beyond that, the proposed RIS provides the filtering effect in the frequency domain, providing a stronger ability to mitigate EMI.

On the other hand, the proposed RIS does show the unwanted loss in the passband. As analyzed in Note 5, the loss is primarily due to the filter chip, which comes at the cost of the sharp frequency selection and high rejection in the stopbands. The insertion loss of the phase shifters and conversion loss between the spatial waves and the guided waves also contribute to the result. To address this issue, a possible solution would be to integrate low-noise amplifiers in the functional modules of the RIS, while ensuring minimal noise is introduced to the signal, as predicted by Ref. [4].

References

[1] QORVO, QPQ3500, https://cn.qorvo.com/products/p/QPQ3500 (2023).

[2] Park, K. & Min, B.-W. Delay-Sum Group Delay Controller with Low-Loss and Low-Phase Variation. *IEEE Transactions on Microwave Theory and Techniques* **69,** 825-832 (2021).

[3] Jeong, J.-C., Yom, I.-B., Kim, J.-D., Lee, W.-Y. & Lee, C.-H. A 6–18-GHz GaAs multifunction chip with 8-bit true time delay and 7-bit amplitude control. *IEEE Transactions on Microwave Theory and Techniques* **66,** 2220-2230 (2018).

[4] Wang, X., et al. Amplification and Manipulation of Nonlinear Electromagnetic Waves and Enhanced Nonreciprocity using Transmissive Space-Time-Coding Metasurface, *Adv. Sci.*, **9**, 2105960 (2022).

[5] Ma, Q. et al. Controllable and Programmable Nonreciprocity Based on Detachable Digital Coding Metasurface. *Advanced Optical Materials* **7**, (2019).

[6] Wu, L., et al. A Wideband Amplifying Reconfigurable Intelligent Surface. *IEEE Transactions on Antennas and Propagation* **70**, 10623–10631 (2022).

[7] https://product.tdk.com/en/search/rf/rf/filter/info?part_no=MMCB2528G5T-0001A3.

[8] Cheng, C.-C. & Abbaspour-Tamijani, A. Study of 2-bit antenna–filter–antenna elements for reconfigurable millimeter-wave lens arrays. *IEEE Trans. Microw. Theory Tech.* **54**, 4498–4506 (2006).

[9] Yang, G. et al. A novel stable miniaturized frequency selective surface, *IEEE Antennas and Wireless Propagation Letters* **9**, 1018-1021 (2010).

[10] Pan, W., Huang, C., Chen, P., Pu, M., Ma, X., & Luo, X. A beam steering horn antenna

using active frequency selective surface. *IEEE Trans. Antennas Propag*. **61**, 6218–6223 (2013).

[11] Reis, J. R. et al. FSS-inspired transmitarray for two-dimensional antenna beamsteering. *IEEE Trans. Antennas Propag*. **64**, 2197–2206 (2016).

[12] Sung, G. et al. A frequency-selective wall for interference reduction in wireless indoor environments, *IEEE Antennas and Propagation Magazine*, **48**, 29-37 (2006).

[13] Shi, Y. et al. Miniaturised frequency selective surface based on 2.5-dimensional closed loop. *Electronics letters* **50**, 1656-1658 (2014).

[14] Poulakis, M. Metamaterials Could Solve One of 6G's Big Problems [Industry View]. *Proceedings of the IEEE* **110**, 1151-1158, (2022).

[15] Di Renzo, M. et al. Smart Radio Environments Empowered by Reconfigurable Intelligent Surfaces: How It Works, State of Research, and The Road Ahead. *IEEE Journal on Selected Areas in Communications* **38**, 2450-2525, (2020).

[16] Björnson, E. et al. Reconfigurable intelligent surfaces: A signal processing perspective with wireless applications. *IEEE Access* **10**, 2646 - 2655 (2021).

[17] Rains, J. High-Resolution Programmable Scattering for Wireless Coverage Enhancement: An Indoor Field Trial Campaign. *IEEE Trans. Antennas Propag*., **71**, 518–530 (2023).

[18] Ali, A. et al. Reconfigurable Intelligent Surface (RIS) in the Sub-6 GHz Band: Design, Implementation, and Real-World Demonstration. *IEEE Access*. **10**, 2169-3536 (2022).

[19] Di Renzo, M. Reconfigurable Intelligent Surfaces vs. Relaying: Differences, Similarities, and Performance Comparison. *IEEE Open Journal of the Communications Society* **1**, 798–807 (2020).

[20] Torres, A., Sanguinetti, L., Björnson, E. Intelligent Reconfigurable Surfaces vs. Decode-and-Forward: What is the Impact of Electromagnetic Interference? arXiv:2203.08046.
